# Preoperative serum total cholesterol is a predictor of prognosis in patients with renal cell carcinoma: a meta-analysis of observational studies

**DOI:** 10.1590/S1677-5538.IBJU.2019.0560

**Published:** 2020-01-10

**Authors:** Bin Li, Deliang Huang, Huilan Zheng, Qiang Cai, Zhenlang Guo, Shusheng Wang

**Affiliations:** 1 Third Clinical Medical College Yangtze University Jingzhou Hospital of Traditional Chinese Medicine Jingzhou China Department of Urology, The Third Clinical Medical College of Yangtze University, Jingzhou Hospital of Traditional Chinese Medicine, Jingzhou, China;; 2 Second Clinical College Guangzhou University of Chinese Medicine Guangzhou China Second Clinical College, Guangzhou University of Chinese Medicine, Guangzhou, China;; 3 Department of Urology Second Affiliated Hospital Guangzhou University of Chinese Medicine Guangzhou China Department of Urology, The Second Affiliated Hospital of Guangzhou University of Chinese Medicine, Guangzhou, China

**Keywords:** Cholesterol, renal cell carcinoma, prognosis, survival, meta-analysis

## Abstract

**Purpose:**

Several studies have demonstrated the strong correlation between the levels of preoperative serum total cholesterol (TC) and the survival of patients with surgically treated renal cell carcinoma (RCC). However, this association remains controversial. We performed a meta-analysis of published reports to evaluate the prognostic significance of the preoperative serum TC levels for patients with surgically treated RCC.

**Material and Methods:**

The databases from MEDLINE (via PubMed), Embase, Web of Science and Cochrane Library were systematically searched to identify the eligible studies published before August 2019. Multivariate adjusted hazard ratios (HRs) with 95% confidence intervals (CIs) were calculated through inverse variance by using random-effects models.

**Results:**

Nine cohort studies comprising 15.609 patients were identified. Low preoperative serum TC levels were associated with poor cancer-specific survival (CSS; HR=0.98, 95% CI: 0.97-0.99; P=0.005; I^2^=74.2%) and progression-free survival (PFS; HR=0.69, 95% CI: 0.49-0.98; P=0.036; I^2^=80%) in patients with surgically treated RCC. However, no significant association was observed between low preoperative serum TC levels and shorter overall survival (HR=0.93, 95% CI: 0.87-1.00; P=0.057; I^2^=86.2%). Sensitivity analyses validated the reliability and rationality of the results.

**Conclusions:**

Preoperative serum TC level is an independent poor prognostic factor for patients with surgically treated RCC, with lower levels associated with worse CSS and PFS. Hence, this parameter may provide additional guidance in the selection of therapeutic strategies to improve prognosis, considering that cholesterol is a broadly applied routine marker in clinical practice.

## INTRODUCTION

Renal cell carcinoma (RCC) has long been the third most common malignancy of the urinary tract and accounts for 2%-3% of adult malignant tumours (1, 2). The incidence of RCC is increasing steadily worldwide, and a recent European study reported 115.000 new RCC cases and 31.000 cancer-related deaths (3, 4). Nephrectomy remains the standard treatment for clinically localised RCC, but 10%-20% of cases may develop metastases (5, 6). The prognosis of advanced RCC after surgery remains poor despite the marked improvement in the survival outcomes of RCC after targeted therapy (7). Thus, appropriate predictors, especially serum biomarkers, are urgently needed to predict the prognosis of RCC.

Cholesterol is mainly synthesized by the liver, and malignant cells require excess cholesterol compared with normal cells (8). Serum high-density lipoprotein, including serum total cholesterol (TC), has been associated with the risk of breast cancer (9). Several clinical risk factors, such as body mass index and nutritional status, have also been considered as potential prognostic factors for RCC (10, 11). However, a comprehensive systematic review or meta-analysis of the association between levels of preoperative serum TC and survival outcomes of patients with surgically treated RCC has not been performed.

Few studies have directly suggested the association between preoperative serum TC levels with worse survival in patients with surgically treated RCC (12-14), but conflicting results have been reported (15). Comprehensive systematic review and meta-analysis are necessary to evaluate the prognostic value of preoperative serum TC levels in patients with surgically treated RCC. The results may be beneficial for treatment selection and postoperative monitoring.

## MATERIALS AND METHODS

Meta-analysis was conducted in accordance with the guidelines of the preferred reporting items for systematic reviews and meta-analyses (PRISMA) (16) and Cochrane Collaboration criterion (17). Thus, ethical approval and patient consent were not required.

### Literature Search

We conducted a comprehensive literature search using the databases of MEDLINE (via PubMed), Embase, Web of Science and Cochrane Library to determine the relevant studies up to August 2019. The combination of medical subject headings (MeSH) and non-MeSH terms, such as ‘renal cell carcinoma’, ‘renal carcinoma’, ‘renal cancer’, ‘kidney cancer’ and ‘cholesterol’ were used, without region, publication type or language restrictions. We also manually searched the reference lists of all original studies recovered and those of previous review articles to identify additional relevant studies. The main search was completed by the senior author, and a professional librarian who directly assisted with the search and confirmed the search terms was recruited. Any discrepancy was resolved by consulting another investigator who was not involved in the initial procedure.

### Study Selection

Two independent investigators screened titles and abstracts to determine eligibility and comprehensively evaluated the full texts of the eligible records in case of uncertainty.

Eligible studies were included if they satisfied the following inclusion criteria: 1) pathologically confirmed diagnosis of RCC, 2) assessed serum TC levels prior to surgery (i.e. radical nephrectomy, cytoreductive nephrectomy and partial nephrectomy), 3) followed a prospective or retrospective study design and 4) directly reported the hazard ratio (HR) with corresponding 95% confidence intervals (CIs) or cases in which the data were available to recalculate risk estimates. If several trials pertained to an overlapping patient population, then the trial with the largest groups of patients was retained (where appropriate) to avoid duplication of information. Disagreements were resolved by consensus between the two investigators.

### Data Extraction and Quality Evaluation

The following data were extracted from all included studies into a standardised Excel (Microsoft Corporation) file: first author name, publication year, study design, research country, cancer site and stage, patient age, sample size and sex proportion, Fuhrman grade, follow-up duration, cut-off value, survival outcome types and HRs of the preoperative serum TC levels for survival outcomes and corresponding 95% CIs. Moreover, we contacted the corresponding authors for additional original data if the eligible records did not provide sufficient information. The accuracy of all extractions was checked by two independent investigators. Disagreements were resolved via a discussion with a third investigator.

The quality of the observational studies was evaluated by two independent investigators using the Newcastle-Ottawa scale (NOS) (18), which consists of nine items to evaluate the representativeness of all included studies. The total score ranged from 0 to 9 and was categorized as follows: a score of 8-9 was considered as high quality, 6-7, moderate quality, 5 or lower, low quality. Disagreements were resolved through a discussion among the authors.

### Statistical Analysis

The prognostic value of different preoperative serum TC levels in patients with surgically treated RCC was assessed on the basis of HRs with corresponding 95% CIs via Stata version 15.0 (serial number: 10699393, StataCorp Wyb). An I^2^ test was conducted to evaluate the heterogeneity of the combined HRs, and significant heterogeneity of I^2^≥50% warranted the use of random-effects models through inverse variance in line with the Cochrane Review guidelines (17). Otherwise, a fixed-effects model was applied. Moreover, pooled HR <1 demonstrated a worse prognosis for patients with lower preoperative serum TC levels, whereas pooled HR >1 suggested better prognosis when P <0.05. The potential factors contributing to heterogeneity were analysed through subgroup analyses stratified by country and tumour type. Sensitivity analyses were conducted by omitting individual study at a time to assess the robustness of the results. Meta-regression analysis was performed to explore the possible sources of heterogeneity in several variables, and restricted maximum likelihood was applied in the analysis. However, the application of Egger (19) and Begg-Mazumdar (20) tests was limited due to the small number of studies evaluated.

## RESULTS

### Literature Search and Study Selection

**Figure 1:**
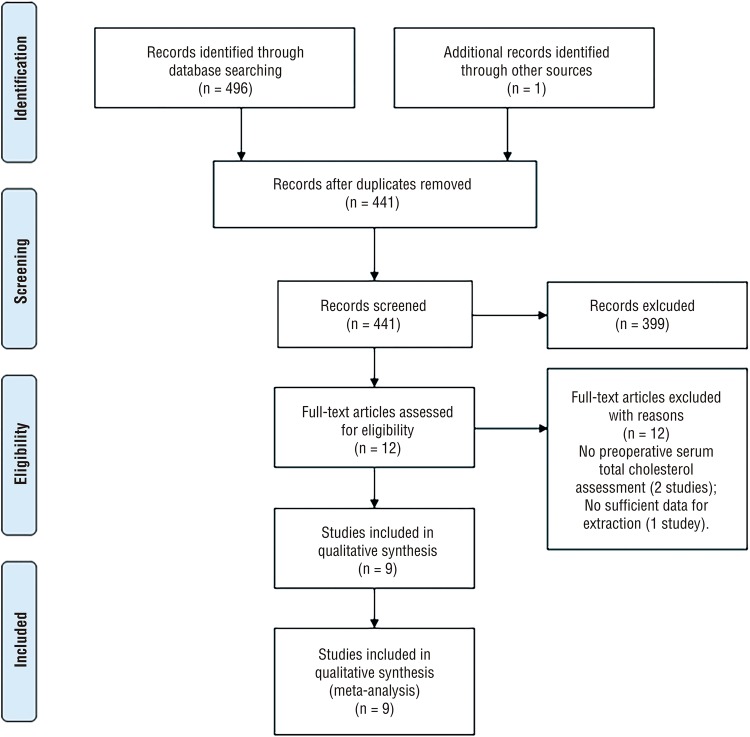
Flow diagram of literature searches according to the Preferred Reporting Items for Systematic Reviews and Meta-Analyses statement.

A total of 497 publications were identified in line with the previously described comprehensive search strategy. Only 411 studies remained after 86 duplicates were removed. After screening the titles and abstracts of the 411 articles, only 12 articles were further assessed via full texts. Among these, three full-text articles were excluded because of the missing preoperative serum TC assessment in two studies and insufficient data for extraction in the other. Thus, nine retrospective cohort studies (12-15, 21-25) that comprised 15.609 patients were included in the meta-analysis. The flowchart of database search and study selection is depicted in Figure-1.

### Study Characteristics and Quality Evaluation

The basic clinical characteristics of the enrolled studies (12-15, 21-25) are provided in Table 1. For the study design, the nine included studies were retrospective cohorts and published from 2014 to 2019. The mean age ranged from 51.3 years to 64 years, and the mean follow-up duration ranged from 13 months to 81.1 months. The enrolled studies were performed in Korea (n=4) (13, 14, 22, 23), China (n=2) (12, 25), Austria (n=1) (21), the United States (n=1) (15) and Japan (n=1) (24). Tumour types at diagnosis and main patient cancer characteristics (i.e. tumour stage and Fuhrman grade) varied. Few studies included only locally or metastatic RCC patients (14, 23), but most studies reported the inclusion of generalized RCC patients (12, 13, 15, 21, 22, 24, 25). Overall survival (OS) was reported in five studies (12-15, 23), cancer-specific survival (CSS) was assessed in six studies (14, 21-25), and progression-free survival (PFS) was evaluated in four studies (12, 14, 15, 25). Finally, HRs and corresponding 95% CIs were sufficiently provided by all included studies.

**Table 1:** Characteristics of the included studies.

First author year	Study design	Country	Tumor type	Sample size (M/F)	Mean age of patients (range)	TNM stage (I/II/III/IV)	Fuhrman grade (I/II/III/IV)	Follow-up (months)	Survival analysis	Cut-off value	Multivariate analysis
de Martino M 2015 (21)	Retrospective (2002-2012)	Austria	RCC	559/ 308	64 (54-72)	T: 503/364 (I–II/III–IV) N: 835/32 (N0/N+) M: 750/117 (M0/M1)	650/217 (I–II/III–IV)	Median 52	CSS	195mg/dL	Yes
Guo S 2016 (12)	Retrospective (2000-2012)	China	RCC	526/ 260	51.3	T: 544/132/83/27 (I/II/III/IV) N: 729/57 (N0/N1) M: 755/31 (M0/M1)	198/278/67/8/235 (unkown)	Median 81.1	OS/PFS	200mg/dL	Yes
Haddad AQ 2015 (15)	Retrospective (2000-2012)	United States	RCC	291/ 217	55 (20-87)	T: 300/197 (I–II/III–IV)	NR	Median 25	OS/PFS	161.5mg/dL	Yes
Jeong HC 2019 (13)	Retrospective (1988-2015)	Korea	nmRCC	2.368/ 1.001	57	T: 2755/614 (I–II/III–IV)	1616/1753 (I–II/III–IV)	Median 38.8	OS	163mg/dL	Yes
Kang HW 2018 (22)	Retrospective (1999-2011)	Korea	RCC	2.205/ 850	55.8	T: 2440/344 (I–II/III–IV)	1547/1468 (I–II/III–IV)	Median 37	CSS	156mg/dL	Yes
Lee H 2017 w(1)(14)	Retrospective (1999-2016)	Korea	lRCC	3.515/ 1.507	56.9	T: 4029/445/527/21 (I/II/III/IV)	2681/2341 (I–II/III–IV)	Median 55	OS/CSS/PFS	161mg/dL	Yes
Lee H 2017 (2)(23)	Retrospective (NR)	Korea	mRCC	185/ 59	59	T: 60/36/117/31 (I/II/III/IV)	34/210 (I–II/III–IV)	Median 13	OS/CSS	170mg/dL	Yes
Ohno Y 2014 (24)	Retrospective (1990-2009)	Japan	CCRCC	273/ 91	60	T: 259/35/66/4 (I/II/III/IV)	278/86 (I–II/III–IV)	Median 71	CSS	150mg/dL	Yes
Peng D 2017 (25)	Retrospective (NR)	China	RCC	952/ 408	55	T: 1015/113/225/7 (I/II/III/IV)	374/738/237/11	Median 67	CSS/PFS	148mg/dL	Yes

The qualities of the included studies were assessed on the basis of NOS. Six studies (12-14, 21, 22, 25) acquired 8 or 9 points and were considered as high-quality reports, and the other three studies (15, 23, 24) acquired 6 or 7 points and were considered of moderate quality. All these studies were included in the meta-analysis.

**Figure 2:**
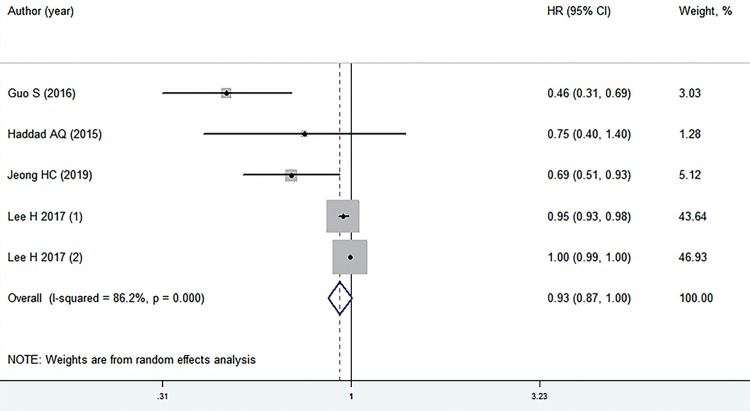
Preoperative serum TC levels and OS in patients with surgically treated RCC. TC = total cholesterol; OS = overall survival; CI = confidence interval; HR = hazard ratio Note: individual studies are represented by a black square and a horizontal line, which corresponds to the point estimate and 95% confidence interval of the odds ratio. The size of the black square reflects the weight of the study in the meta-analysis. The solid vertical line corresponds to ‘no effect’ of treatment-an hazard ratio of 1. The diamond at the bottom and dotted line represent the combined or pooled hazard ratio of all five trials with its 95% confidence interval.

Five retrospective cohort studies comprising 9.929 patients reported the OS (12-15, 23). The results indicated no association between low preoperative serum TC levels and OS (HR=0.93, 95% CI: 0.87-1.00; P=0.057; I^2^=86.2%) (Figure 2). When stratified by different countries, the results were inconsistent with the overall analysis except for the study performed in China (12) because of the limited number of studies evaluated. In the tumor type subgroup, low preoperative serum TC levels were significantly associated with worse OS of RCC, but not for patients with metastatic and localized RCC, except for the OS in the metastatic RCC group (Table-2) (23). In addition, the results of sensitivity analyses demonstrated the strength of our findings by omitting each single study in turn (Table-3). Finally, meta-regression analyses were conducted to further explore the significant heterogeneity among the studies, and the results indicated that none of the covariates (country, P=0.889, tumour type, P=0.969) resulted in heterogeneity.

**Table 2:** Results of subgroup analyses.

OS	Studies, N	Participants,N	HR (95% CI)	p value	p of heterogeneity	I2 (%)

	5	9.929	0.93 (0.87-1.00)	0.057	<0.001	86.2
**Country**						
China	1	786	0.46 (0.31-0.69)	<0.001	NA	NA
United States	1	508	0.75 (0.40-1.40)	0.368	NA	NA
Korea	3	8.635	0.97 (0.92-1.02)	0.187	0.001	85.8
**Tumor type**						
RCC	2	1.294	0.55 (0.35-0.88)	0.012	0.197	39.8
nmRCC	1	3.369	0.69 (0.51-0.93)	0.015	NA	NA
lRCC	1	5.022	0.95 (0.92-0.98)	0.001	NA	NA
mRCC	1	244	1.00 (0.99-1.01)	0.076	NA	NA
**CSS**	**Studies, N**	**Participants, N**	**HR (95% CI)**	**p value**	**p of heterogeneity**	**I**^**2**^ **(%)**
	6	10.946	0.98 (0.97-0.99)	0.005	0.002	74.2
**Country**						
Austria	1	867	0.94 (0.91-0.98)	0.001	NA	NA
Korea	3	8.321	0.99 (0.99-1.00)	0.014	0.144	48.4
Japan	1	364	0.99 (0.98-1.00)	0.009	NA	NA
China	1	1.360	0.59 (0.40-0.88)	0.009	NA	NA
**Tumor type**						
RCC	3	5.282	0.95 (0.89-1.02)	0.181	0.001	86.6
lRCC	1	5.022	0.96 (0.92-0.99)	0.018	NA	NA
mRCC	1	244	0.99 (0.99-1.00)	0.023	NA	NA
CCRCC	1	364	0.99 (0.98-1.00)	0.009	NA	NA

**CCRCC =** clear cell renal cell carcinoma; **CI =** confidence interval; **CSS =** cancer-specific survival; **HR =** hazard ratio; **lRCC =** localised renal cell carcinoma; **mRCC =** metastatic renal cell carcinoma; **nmRCC =** non-metastatic renal cell carcinoma; NA = not available; **OS =** overall survival; **RCC =** renal cell carcinoma

**Table 3:** Results of sensitivity analyses.

Study omitted	HR	95% CI	
**OS**			
Guo S 2016 (12)	0.96	0.91	1.02
Haddad AQ 2015 (15)	0.93	0.87	1.01
Jeong HC 2019 (13)	0.95	0.89	1.02
Lee H 2017 (1) (14)	0.72	0.49	1.05
Lee H 2017 (2) (23)	0.71	0.50	1.01
Combined	0.93	0.87	1.00
**CSS**			
de Martino M 2015 (21)	0.99	0.98	1.00
Kang HW 2018 (22)	0.98	0.96	1.00
Lee H 2017 (1) (14)	0.99	0.98	1.00
Lee H 2017 (2) (23)	0.98	0.96	0.99
Ohno Y 2014 (24)	0.98	0.97	1.00
Peng D 2017 (25)	0.99	0.98	1.00
Combined	0.98	0.97	0.99

**CI =** confidence interval; **CSS =** cancer-specific survival; **HR =** hazard ratio; **OS =** overall survival

Preoperative serum TC levels and CSS and PFS in patients with surgically treated RCC.

**Figure 3:**
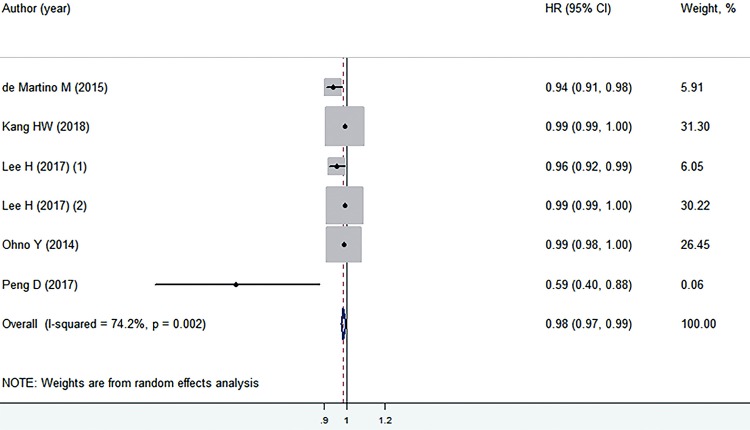
Preoperative serum TC levels and CSS in patients with surgically treated RCC. TC = total cholesterol; OS = overall survival; CI = confidence interval; HR = hazard ratio Note: individual studies are represented by a black square and a horizontal line, which corresponds to the point estimate and 95% confidence interval of the odds ratio. The size of the black square reflects the weight of the study in the meta-analysis. The solid vertical line corresponds to ‘no effect’ of treatment-an hazard ratio of 1. The diamond at the bottom and dotted line represent the combined or pooled hazard ratio of all six trials with its 95% confidence interval.

Six retrospective cohort studies comprising 10.946 patients provided the CSS (14, 21-25). Low preoperative serum TC levels yielded a worse CSS (HR=0.98, 95% CI: 0.97-0.99; P=0.005) in patients with surgically treated RCC, and significant heterogeneity was observed (I^2^=74.2%). Therefore, a random-effects model was applied for the analysis (Figure 3). In the subgroup analysis according to different countries, low preoperative serum TC levels were associated with poor CSS in studies conducted in Austria (21) and China (25), whereas no significant association was observed in studies conducted in Korea (14, 22, 23) and Japan (24). Regarding the tumour type, significant association was observed in patients with localized RCC, but negative results were found in those with RCC, metastatic RCC, and clear-cell RCC (Table-2). The sensitivity analyses indicated that the stability of results exhibited no significant change after each single study was deleted individually (Table-3). The meta-regression analyses revealed that none of the covariates (country, P=0.908, tumor type, P=0.698) resulted in significant heterogeneity among the included studies.

**Figure 4:**
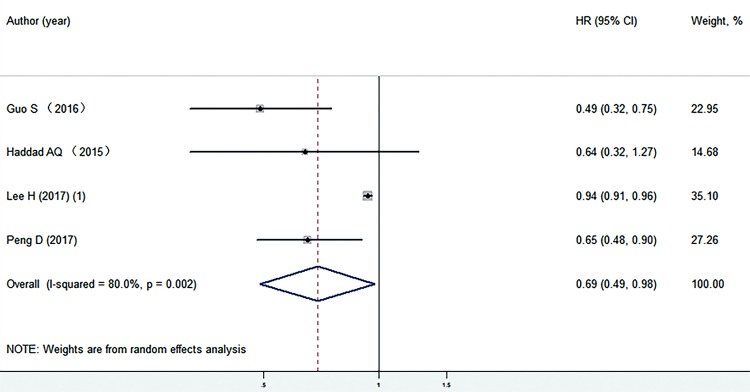
Preoperative serum TC levels and PFS in patients with surgically treated RCC. TC = total cholesterol; OS = overall survival; CI = confidence interval; HR = hazard ratio Note: individual studies are represented by a black square and a horizontal line, which corresponds to the point estimate and 95% confidence interval of the odds ratio. The size of the black square reflects the weight of the study in the meta-analysis. The solid vertical line corresponds to ‘no effect’ of treatment-an hazard ratio of 1. The diamond at the bottom and dotted line represent the combined or pooled hazard ratio of all four trials with its 95% confidence interval.

In the four studies (12, 14, 15, 25) involving 7.676 patients, low preoperative serum TC levels were significantly associated with shorter PFS (HR=0.69, 95% CI: 0.49-0.98; P=0.036, I^2^=80%) (Figure-4). Considering that the number of eligible studies that assessed PFS was relatively small, we did not perform subgroup analysis for PFS.

## DISCUSSION

### Main findings

The meta-analysis included nine retrospective cohort studies that associated preoperative serum TC levels with survival outcomes in patients with surgically treated RCC. The results indicated that low preoperative serum TC levels were significantly associated with worse CSS and PFS, whereas no significant difference was observed in the OS for patients with surgically treated RCC. For the subgroup analysis stratified by country and tumour type, the results were inconsistent with the overall analysis because of the limited number of studies evaluated. Nonetheless, sensitivity analyses indicated that the robustness of the results had no significant change after omitting each single study. Thus, the rationality and reliability of our analysis were validated. Finally, the meta-regression could not identify potential factors that might have substantially affected the heterogeneity between studies.

Most of the enrolled studies generally suggested an increased risk of shortened OS, CSS and PFS in patients with surgically treated RCC, whereas two studies yielded conflicting results (15, 23). Haddad AQ et al. (15) performed a retrospective cohort study comprising 508 patients with surgically treated RCC in the United States. The results showed no association between preoperative serum TC levels and OS or PFS. Lee H et al. (23) demonstrated that a low preoperative serum TC level is not an independent poor prognostic factor for patients with surgically treated RCC, particularly for OS and CSS. Moreover, our results revealed that low preoperative serum TC levels yielded a worse CSS from six retrospective cohort studies, whereas no significant association was observed in three of the studies. A HR of 0.98 and p=0.005 have certain clinical utility, although the HR is close to 1. For instance, Ohno et al. (24) analyzed 364 subjects with clear cell RCC patients and concluded that low preoperative serum TC levels was associated with worse CSS, although the findings of their multivariate analysis were not statistically significant due to a limited number of cases. Hence, it is necessary to expand the sample size for more statistical power in the future study. Of note, when these studies were discarded from the meta-analysis, the results showed no significant changes, thus validating the reliability of our results.

### Implications for clinical practice

Cholesterol is a crucial part of the human cell membrane. This molecule is an important substitute for energy production and controlled by the feedback adjustment system and maintains homeostasis (26). A high serum TC level has been recently considered a poor prognostic predictor of cardiovascular disease and stroke. Thus, controlling the cholesterol level is an important issue for most clinicians. RCC is a metabolic disease with many risk factors, such as obesity, diabetes mellitus and nutritional status, which are closely associated with high risk of RCC or its prognosis (27-30). Histologically, a high level of cholesterol and glycogen accumulates in the clear-cell RCC (31). Saito K et al. studied 356 lipids and observed a significant difference in more than 70% of lipid levels. They distinguished the lipid profiles of RCC tissue from the clear-cell RCC tissue with normal parenchymal tissue (32). In addition, the lower serum TC levels in patients with advanced RCC may have been caused by increased storage of cholesterol in the cancer cells, and its application in the biosynthesis of new membrane has been suggested (33). However, whether serum TC can be used as a substitute for tumour cholesterol and whether these serum TC levels are pre-existing or tumour-causing remain unclear. Future studies should focus on the aforementioned complex relationship and address these interesting issues. The assessment of preoperative serum TC levels may provide a meaningful adjunct in clinical practice because cholesterol is a broadly applied routine marker. Our findings will help clinicians identify patients who suffer from RCC, undergo surgery and have a high risk of poor postoperative outcomes. Our findings will also aid in determining personalised therapeutic strategies after RCC surgery.

### Strengths and limitations

To the best of our knowledge, our meta-analysis is the first to deeply investigate the association between preoperative serum TC levels and survival outcomes in patients with surgically treated RCC in accordance with the PRISMA guideline. Moreover, multivariate-adjusted risk estimates were applied to minimize other relevant confounding factors that might affect the overall results. Finally, the results of sensitivity analyses and meta-regression validated the reliability and rationality of our meta-analysis.

This study had several limitations that should be considered. Firstly, only nine studies comprising 15.609 patients were included, and the sample size was relatively small. Secondly, the heterogeneity between the studies might affect overall results, although the meta-regression could not identify the potential factors. Thirdly, all included studies applied a retrospective design with disadvantages on potentially missing data and risk of bias. Finally, the included studies had diverse cut-off values which may affect the utility of our results. A unified cut-off value of preoperative serum TC should be established. Further investigations regarding such an association in large-scale studies with greater statistical power are needed.

## CONCLUSIONS

In summary, preoperative serum TC levels is an independent prognostic predictor for patients with surgically treated RCC. Lower levels of this parameter were associated with worse CSS and PFS. Hence, the assessment of preoperative serum TC levels may be beneficial to stratify the risk and individualize treatment for patients with surgically treated RCC to improve prognosis. Further large-scale investigations on such association with greater statistical power are still required to confirm our findings.
